# DNA methylation of *microRNA-375* in impaired glucose tolerance

**DOI:** 10.3892/etm.2014.1816

**Published:** 2014-06-30

**Authors:** XIAOLI WANG, XIANGYUN CHANG, JUN LI, LIANG YIN, KAN SUN

**Affiliations:** Departments of Endocrinology and Metabolism, The First Affiliated Hospital, Shihezi University School of Medicine, Shihezi, Xinjiang 832002, P.R. China

**Keywords:** type 2 diabetes, impaired glucose tolerance, *miR-375*, methylation

## Abstract

In the present study, the expression levels and DNA methylation status of *microRNA* (*miRNA*)-375 in patients with impaired glucose tolerance (IGT) and type 2 diabetes mellitus (T2DM) were analyzed and the role of DNA methylation of *miRNA-375* in the pathogenesis of T2DM was investigated. Compared with the *miR-375* levels in patients with normal glucose tolerance (NGT; n=53), the samples from patients with IGT (n=44) exhibited downregulation of *miR-375*, while those from patients with T2DM (n=54) exhibited upregulation of *miR-375* in the plasma. Additionally, the samples from patients with IGT were observed to be hypermethylated compared with those from patients with T2DM and NGT (P=0.042). Analysis of three CpG units (CpG1.2, CpG20 and CpG25.26.27) from 17 CpG sites (between −990 and −1,258 bp, relative to the transcription start site) revealed higher methylation levels in patients with IGT compared with those in patients with NGT (P<0.05). The methylation of two CpG units (CpG1.2 and CpG25.26.27) was higher in patients with IGT than in the patients with T2DM (P<0.05). Thus, the present study demonstrated that the *miR-375* promoter was hypermethylated and the levels of *miR-375* in the plasma were downregulated in the patients with IGT. DNA hypomethylation may have an important role in the regulation of *miR-375* expression and may contribute to the pathogenesis of T2DM.

## Introduction

Type 2 diabetes mellitus (T2DM) and impaired glucose tolerance (IGT) results from an interaction between genetic and environmental factors (1). Current evidence favors a two-step development of T2DM (2–5). During step one, individuals with normal glucose tolerance (NGT) progress to IGT with insulin resistance as the primary determinant. In step two, IGT advances to T2DM as a result of a progressive decline in β-cell function (1–3,6). The genetic background causes insulin resistance and β-cell failure. Polymorphisms in genes that are involved in insulin secretion have been identified, and responses may modify individual disease susceptibility; however, in large population-based studies only a few polymorphisms in these genes have been shown to influence the incidence of diabetes (7–9).

microRNAs (miRNAs) have been implicated in the pathogenesis of numerous human diseases (10). There is increasing evidence that miRNAs are also involved in the pathogenesis of metabolic diseases, including diabetes mellitus. However, few miRNAs have been investigated in pancreatic β cells (11–15). miRNAs are important for β-cell development, as deletion of the enzyme Dicer results in a severe loss of these cells (16). *miR-375* is one of the most abundant miRNAs in β cells (11) and is necessary for their proper development and maintenance. However, overexpression of *miR-375* suppresses glucose-induced insulin secretion, and conversely, inhibition of endogenous *miR-375* function enhances insulin secretion, suggesting that *miR-375* is a negative regulator of β-cell exocytosis (11). Despite the apparent importance of this miRNA, the regulation of *miR-375* remains poorly understood.

A study demonstrated that there is an important link between methylation, gene dosage effects, and diabetes (17). Methylation has an important role in regulating gene expression, including the expression of genes essential for the strict maintenance of normal blood glucose levels. *miR-375* is located in an intergenic region and has an independent promoter containing CpG islands. Since CpG islands are the structural basis for regulation by methylation, it was hypothesized in the present study that differential expression and CpG methylation of *miR-375* may have a role in the development of IGT and T2DM.

In this study, changes in *miR-375* expression were investigated and the quantitative methylation status of CpG islands within the *miR-375* promoter was measured to determine whether aberrant promoter methylation of *miR-375* occurred in NGT, IGT and T2DM, and whether the patterns of methylation affect *miR-375* expression.

## Materials and methods

### Patients

From 2010 to 2012, data were collected from the Departments of Endocrinology and Metabolism at Shihezi University School of Medicine (Shihezi, China). Patients with T2DM (n=54), IGT (n=44) and NGT (n=53, as controls) were recruited in this study. Patients with T2DM (28 men and 26 women, mean age 52.9±9.7 years) had been hospitalized for treatment of poor glucose control. Patients with IGT (23 men and 21 women, mean age 54.3±8.6 years) and control patients (23 men and 30 women, mean age 52.9±9.4 years) were recruited from the patients who underwent health examinations at the First Affiliated Hospital, Shihezi University School of Medicine. All patients underwent a standard oral glucose tolerance test, as recommended by the American Diabetes Association. Diagnosis of T2DM and IGT were based on the World Health Organization criteria (1999) (18). Any patient suspected of having any infectious disease shortly prior to or during the study was excluded from study, as were patients with autoimmune diseases. All patients gave informed written consent prior to the start of the study. This study was conducted in accordance with the principles of the Declaration of Helsinki. The present study was approved by the ethics committee of the Shihezi university.

### Nucleic acid isolation

RNA was isolated from plasma samples using the miRNeasy Mini kit (Cat. no. 217004; Qiagen, Valencia, CA, USA) and was quantified using absorption measurements at 260 nm (Toption Instrument Co., Ltd, Xi’an, China). Genomic DNA was isolated using the DNeasy Blood and Tissue kit (Qiagen) and was quantified spectrophotometrically at 260 nm (Toption Instrument Co., Ltd).

### Quantitative polymerase chain reaction (qPCR)

qPCR was performed using an ABI Prism 7500 Fast Real-time PCR system (Applied Biosystems, Foster City, CA, USA), Taqman Universal PCR Master mix (Applied Biosystems), a Taqman Reverse Transcription kit (Applied Biosystems), Taqman MicroRNA assays (Applied Biosystems), and Human Panel Early Access kit (Applied Biosystems) in accordance with the manufacturer’s instructions. Expression levels of miRNAs were based on the amount of the target message relative to that of the *microRNA-16* transcript as a control to normalize the initial input of total RNA. PCR was performed under the following conditions: 50°C for 2 min then 95°C for 10 min, followed by 40 cycles at 95°C for 15 sec and 60°C for 1 min.

### Sequenom methylation analysis

To quantify the methylation levels of the *miR-375* CpG islands in clinical samples, the high-throughput MassARRAY platform (Sequenom, Inc., San Diego, CA, USA) was used. Briefly, bisulfite-treated DNA was amplified with primers for the *miR-375* CpG islands. The primers were designed using EpiDesigner (Sequenom Inc.) and were as follows: Forward 5′-aggaagagagGGGTGGAGTATTTTTGTTTGTTG-3′ and reverse 5′-cagtaatacgactcactatagggagaaggctAAAAACATAATCCAAAACATCCTAAT-3′. The PCR products were spotted on a 384-pad SpectroCHIP (Sequenom, Inc.), followed by spectral acquisition on a MassARRAY Analyzer (Sequenom, Inc.). Methylation data of individual units (1–3 CpG sites per unit) were generated using EpiTyper v1.0.5 software (Sequenom, Inc.).

### Statistical analysis

Data are presented as the mean ± standard deviation. To compare the mean of more than two groups, analysis of variance was used. The χ^2^ test was used for comparisons of numeration data. The expression of *miR-375* was calculated using the 2^−ΔΔCt^ method (19). Since the data for *miR-375* expression and DNA methylation were not normally distributed and exhibited heterogeneous variance, the Kruskal Wallis test was used. P<0.05 was considered to indicate a statistically significant difference.

## Results

### Patient characteristics

Table I presents the anthropometric and metabolic characteristics of the study groups. Patients with IGT were slightly more obese than patients with NGT. For patients with T2DM, abdominal obesity was greater compared with that of patients with IGT and NGT, and patients with T2DM had higher triglyceride concentrations. Additionally, patients with T2DM and IGT had lower high-density lipoprotein cholesterol (HDL-C) levels compared with those in patients with NGT.

### miR-375 expression in T2DM, IGT and NGT samples

**Figure 1:**
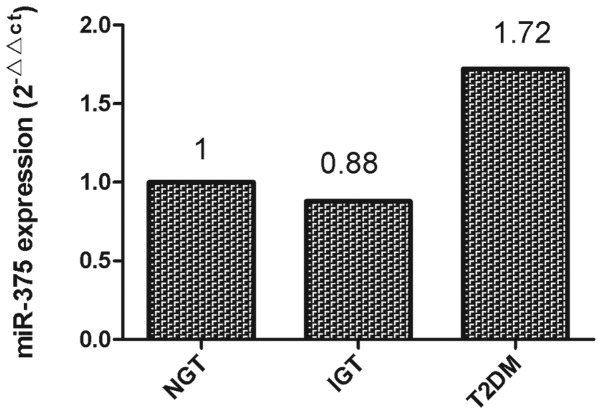
*miR-375* expression in T2DM, IGT and NGT samples was detected using quantitative polymerase chain reaction. The value is defined as the expression ratio of *miR-375* to *miR-16*. T2DM, type 2 diabetes mellitus; IGT, impaired glucose tolerance; NGT, normal glucose tolerance.

qPCR was performed to investigate the expression of *miR-375* in plasma samples from patients with T2DM, IGT and NGT, respectively. As shown in Fig. 1, downregulation of plasma *miR-375* levels was detected in the samples from the patients with IGT (0.88 fold of NGT), whilst upregulation of plasma *miR-375* was detected in the samples from the patients with T2DM (1.72 fold of NGT).

### DNA methylation of miR-375

**Figure 2:**
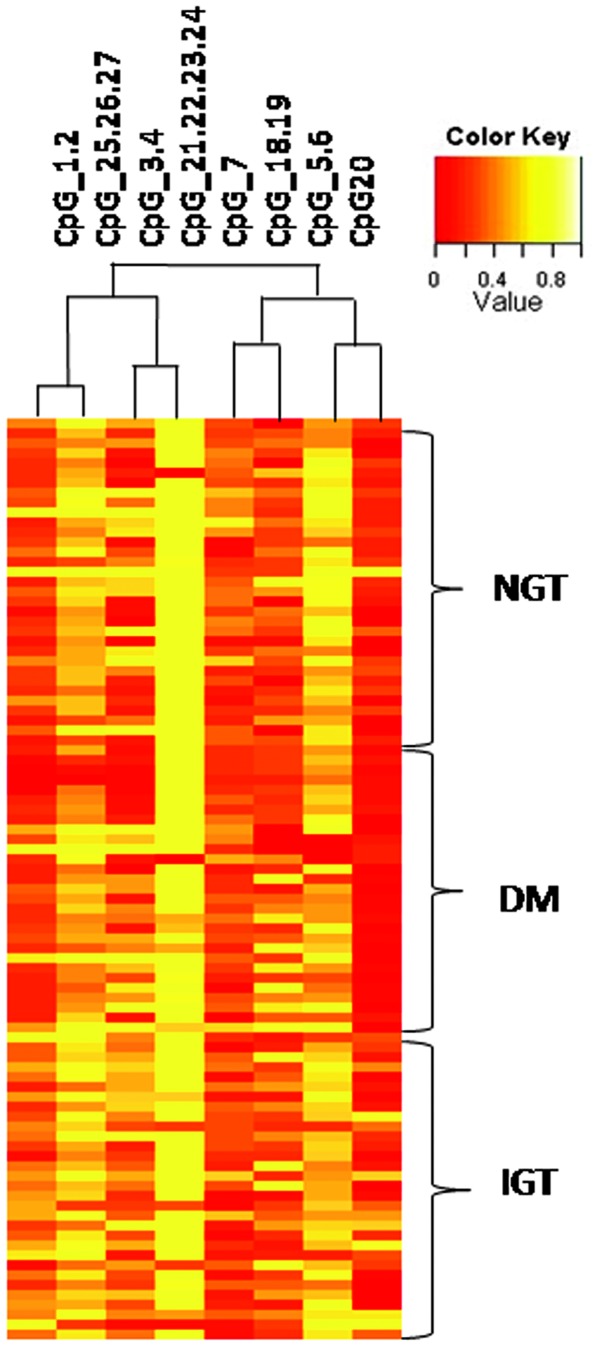
Hierarchical clustering of *miR-375* methylation profiles from IGT, T2DM and NGT samples was determined using MassARRAY analysis. Each row represents a sample. Each column represents a CpG unit, defined as a single CpG site or a combination of CpG sites. Color coding reflects the degree of methylation, with yellow being 100% and red being 0%. T2DM, type 2 diabetes mellitus; IGT, impaired glucose tolerance; NGT, normal glucose tolerance.

In order to understand the mechanism of *miR-375* upregulation, the methylation status in the promoter region of *miR-375* was investigated. In total, 44 IGT, 54 T2DM and 53 NGT samples were analyzed using MassARRAY. Hierarchical clustering identified differences in the quantitative methylation profiling of IGT cases compared with T2DM and controls (Fig. 2).

**Figure 3:**
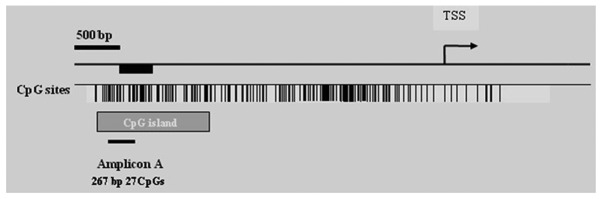
Map of the *miR-375* promoter region. Vertical lines depict CpG dinucleotides. The arrow indicates the transcriptional start site. Vertical bars: CpG sites; gray filled box: CpG island; black filled bars, amplicons studies by MassARRAY. Amplicon characteristics are shown beneath the black bars. bp, base pair; TSS, transcriptional start site.

**Figure 4:**
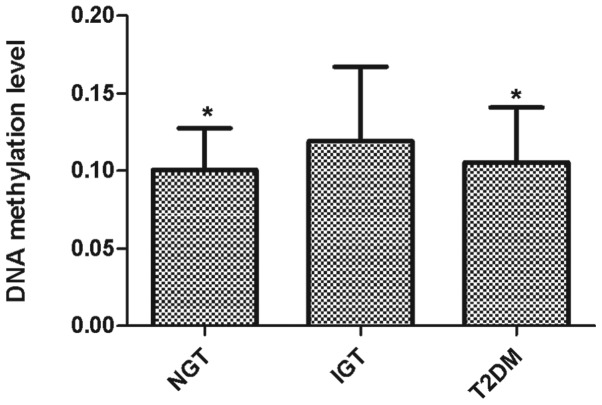
DNA methylation levels of T2DM, IGT and NGT samples. ^*^P<0.05, compared with IGT. The error bars represent standard errors. T2DM, type 2 diabetes mellitus; IGT, impaired glucose tolerance; NGT, normal glucose tolerance.

**Figure 5:**
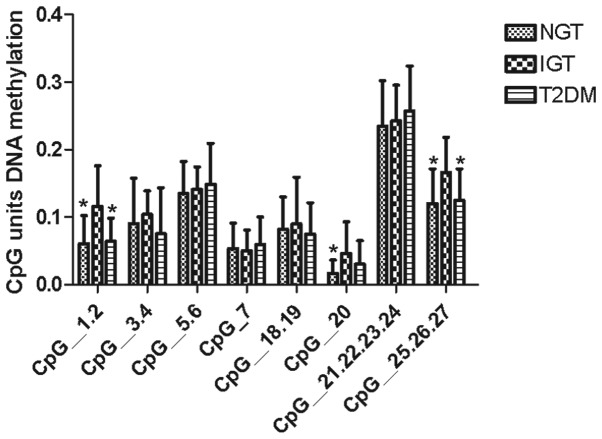
Comparison of *miR-375* methylation in T2DM, IGT and NGT samples. The average methylation of the CpG units of amplicons in T2DM, IGT and NGT samples is shown. ^*^P<0.05, compared with IGT. The error bars represent standard errors. T2DM, type 2 diabetes mellitus; IGT, impaired glucose tolerance; NGT, normal glucose tolerance.

*miR-375* methylation was assessed from bp −990 to bp −1258 relative to the transcription start site (Fig. 3). Eight CpG units, incorporating 17 CpG residues spanning 267 bp on the specified promoter region of *miR-375* were analyzed. The mean level of *miR-375* methylation in the plasma samples, calculated from the methylation levels of the 17 CpG residues, was 10.56% for the patients with T2DM, 11.92% for the IGT group and 10.05% for the NGT group. The DNA methylation level in the IGT group was higher than those in the T2DM and NGT groups (P=0.042; Fig. 4). Furthermore, the individual CpG units in T2DM, IGT and NGT cases were analyzed and three specific CpG units (CpG1.2, CpG20, and CpG25.26.27) were found to be hypermethylated in IGT samples compared with the methylation levels in T2DM and NGT samples (Fig. 5).

### Correlation between methylation of CpG units and clinical features

**Figure 6:**
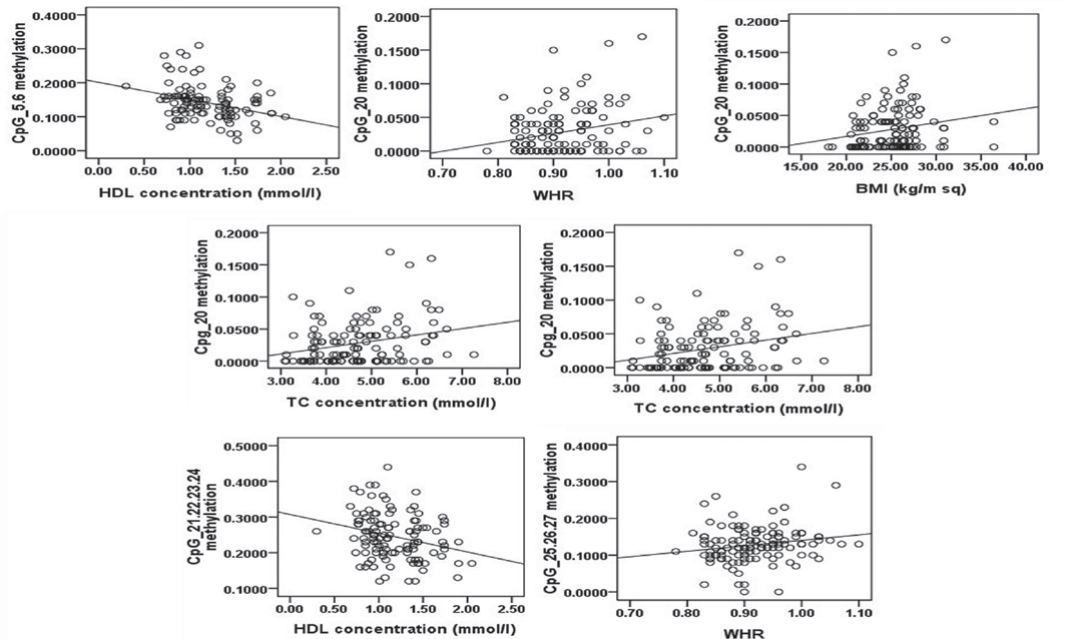
Correlations between methylation of CpG units and clinical features. HDL, high-density lipoprotein; WHR, waist-hip ratio; BMI, body mass index; TC, total cholesterol.

Methylation patterns were then used to investigate the potential correlation with clinical features (Table II). The results showed that none of the clinical parameters were significantly different according to the methylation status of the *miR-375* promoter. However, analysis of eight CpG units demonstrated that methylation of CpG5.6 and CpG21.22.23.24 were negatively correlated with HDL, methylation of CpG20 was positively correlated with body mass index (BMI), waist-hip ratio (WHR), total cholesterol (TC) and low-density lipoprotein (LDL), and methylation of CpG25.26.27 was positively correlated with WHR (Fig. 6).

## Discussion

Recent advances in the understanding of the genetics of T2DM susceptibility have focused on the regulation of transcriptional activity within pancreatic β cells. miRNAs have been demonstrated to have an important role in the control of glucose homeostasis; *miR-375*-null mice are hyperglycemic and exhibit reduced β-cell mass, and the knockdown of *miR-375* in obese ob/ob mice results in a significant effect on glycemia, leading to a severe diabetic phenotype (20). In the present study, the plasma levels of *miR-375* were found to be significantly upregulated in samples from patients with T2DM, but slightly downregulated in samples from patients with IGT compared with those in patients with NGT. A study has shown that the overexpression of *miR-375* suppresses glucose-induced insulin secretion whereas inhibition of endogenous *miR-375* function enhances insulin secretion (11). In β-cell line cultures, *miR-375* inhibits insulin secretion in part by inhibiting the translation of the mRNA for myotrophin (11,21) and phosphoinositide 3-kinase-dependent-kinase (22). This suggests that *miR-375* may be involved in the pathogenesis of IGT and T2DM. During the initial stages of IGT, β-cell function may be enhanced by downregulation of *miR-375* as a compensatory mechanism for insulin resistance. The upregulation of *miR-375*, which suppresses insulin secretion, may be attributed to the progression of IGT followed by T2DM.

Epigenetic modification of DNA, including methylation and/or histone modification, is considered to have an important role in the regulation of DNA expression. Studies have revealed that epigenetics regulates *miR-375* in a number of different types of cancer, including hepatocellular, gastric and breast cancer (23,24). Our previous study has demonstrated that miR-375 promoter was hypomethylated in patients with T2DM compared with the NGT sample (25). In the present study, MALDI-TOF MS (via the MassARRAY analysis) was used to analyze the methylation patterns at multiple CpG sites within the promoter regions of *miR-375*. The results demonstrated hypermethylation patterns in IGT compared with T2DM and NGT. The aberrant methylation status of the CpG units was then investigated. The results showed significant differences in the frequency of methylation at individual CpG units in IGT, T2DM and NGT samples. Three CpG units (CpG1.2, CpG20 and CpG25.26.27) showed higher methylation frequencies in IGT samples than in NGT samples. The methylation of two CpG units (CpG1.2 and CpG25.26.27) was higher in IGT samples than in T2DM samples. The results suggest that *miR-375* CpG island methylation was negatively correlated with *miR-375* expression. Hypermethylation of the *miR-375* promoter may have a key role in the downregulation of its expression in patients with IGT. Compared with the samples from patients with IGT, those from patients with T2DM presented relative hypomethylation of the *miR-375* promoter and upregulation of *miR-375* expression. This suggests there may be demethylation during the course of IGT progression to T2DM.

DNA methylation was originally considered stable and irreversible. However, studies have shown that environmental factors influence the regulation of DNA methylation in mammals (26–28). In the present study, the potential correlation of methylation patterns with clinical features was investigated. The results demonstrated that BMI, WHR, LDL and TC were positively correlated with DNA methylation. Increased body weight has been reported to be an important factor for DNA methylation patterns (29). Acute exposure to the free fatty acids palmitate and oleate has been demonstrated to increase the promoter methylation of genes involved in mitochondrial functioning in human primary muscle cells (30). However, no direct evidence has shown that hyperlipidemia influences DNA methylation; therefore, further studies are required to fully elucidate the mechanisms involved in this phenomenon.

In conclusion, in the present study, the hypermethylation status of the *miR-375* promoter and the downregulation of plasma levels of *miR-375* in patients with IGT were described. The results suggest that DNA hypomethylation may have a role in the regulation of *miR-375* expression and may contribute to the pathogenesis of T2DM.

## Figures and Tables

**Table I:** Anthropometric and metabolic characteristics of the study groups.

Parameter	T2DM	IGT	NGT	F-value	P-value
n	5444	53-	-		
Gender (male/female)	28/2623/21	23/30-	-		
Age (years)	52.9±9.7	54.3±8.6	52.9±9.4	0.360	0.699
SBP (mmHg)	135±15	135±15	133±16	0.493	0.612
DBP (mmHg)	77±9	78±9	79±10	0.550	0.578
BMI (kg/m^2^)	25.28±2.79	25.71±1.14	24.21±3.89^a^	3.519	0.032
WHR	0.92±0.06^a^,^b^	0.96±0.06^b^	0.88±0.05	23.110	<0.001
FBG (mmol/l)	8.52±2.90^a^,^b^	6.29±0.52^b^	5.11±0.67	47.348	<0.001
Fins (mU/l)	40.65±27.02	39.65±22.23	50.75±40.51	2.532	0.083
FCP (mmol/l)	0.79±0.36^a^^b^	1.19±0.53^b^	1.51±0.59^a^	28.262	<0.001
HbA1c (%)	8.56±1.75^a^^b^	5.60±0.65^b^	4.91±0.50	150.147	<0.001
TG (mmol/l)	2.16±2.10^b^	1.84±1.09	1.56±1.02	2.150	0.120
TC (mmol/l)	4.75±0.90	4.84±1.01	4.54±0.77	1.463	0.235
LDL (mmol/l)	2.83±0.74	3.06±0.97	2.62±0.69	3.632	0.029
HDL (mmol/l)	1.10±0.28^b^	1.10±0.29^b^	1.29±0.34	6.464	0.002

Data are presented as the mean ± standard deviation. T2DM, type 2 diabetes mellitus; IGT, impaired glucose tolerance; NGT, normal glucose tolerance; SBP, systolic blood pressure; DBP, diastolic blood pressure; BMI, body mass index; WHR, waist-hip ratio; FBG, fasting blood glucose; Fins, fasting insulin; FCP, fasting plasma C-peptide; HbAlc, glycated hemoglobin; TG, triglycerides; TC, total cholesterol; LDL, low-density lipoprotein; HDL, high-density lipoprotein.

a P<0.05, compared with IGT;

b P<0.05, compared with NGT.

**Table II:** Correlation between methylation of CpG units and clinical features (R values)

CpG unit	Age	BMI	WHR	SBP	DBP	HbA1C	Fins	FCP	TG	TC	LDL	HDL	FBG
CpG5.6	0.082	0.126	0.120	0.162	0.114	0.065	0.077	0.007	0.185	0.084	0.106	−0.328^a^	0.143
CpG20	−0.080	0.199^a^	0.244^b^	0.065	0.101	0.132	−0.073	−0.163	0.015	0.263^b^	0.299^b^	−0.022	0.154
CpG21.22.24.24	−0.030	0.016	0.016	0.113	0.119	0.107	−0.045	−0.055	0.153	0.001	0.056	−0.264^b^	0.122
CpG25.26.27	0.026	0.095	0.186^a^	0.089	0.118	−0.044	0.112	0.086	0.056	−0.055	−0.048	−0.060	0.068

a P<0.05,

b P < 0.01.

BMI, body mass index; WHR, waist-hip ratio; SBP, systolic blood pressure; DBP, diastolic blood pressure; HbA1C, glycated hemoglobin; Fins, fasting insulin; FBG, fasting blood glucose; FCP, fasting plasma C-peptide; TG, triglycerides; TC, total cholesterol; LDL, low-density lipoprotein; HDL, high-density lipoprotein.
